# A Review on the Use of Confocal Laser Endomicroscopy in the Bile Duct

**DOI:** 10.1155/2012/454717

**Published:** 2012-04-19

**Authors:** Ioana Smith, Pamela E. Kline, Monica Gaidhane, Michel Kahaleh

**Affiliations:** ^1^Department of Medicine, University of Virginia Health System, Charlottesville, VA 22908, USA; ^2^Department of Medicine, Weill Cornell Medical College, New York, NY 10065, USA; ^3^Division of Gastroenterology and Hepatology, Weill Cornell Medical College, New York, NY 10021, USA

## Abstract

*Background*. Current methods to diagnose malignant biliary strictures are of low sensitivity. Probe-based confocal laser endomicroscopy (pCLE) is a new approach that can be used to evaluate in vivo histopathology of the GI tract. This paper is of studies evidencing pCLE's application in the diagnosis of indeterminate biliary strictures. *Methods*. This paper examined peer-reviewed studies conducted between January 2000 and November 2011. A PubMed search for relevant articles was performed using the following keywords:“pCLE”, “confocal”, “endomicroscopy”, “probe-based confocal laser endomicroscopy”, “and “bile duct”. Further individual review was done to assess the screened articles' relevance to the topic. *Results*. After individual review, 6 studies were included; with a cumulative sample size of 165, with 75 subjects identified as having a malignancy. These studies included tertiary care centers in Germany, France, and USA, including one multicenter trial. 3 studies assessed pCLE's specificity (range 67%–88%) ,sensitivity (range 83%–98), and accuracy (range 81%–86%). *Conclusion*. Confocal endomicroscopy is a novel and promising modality for the biliary tree. Further studies need to be conducted both to establish its usefulness for the diagnosis of indeterminate biliary strictures and to understand the histological meaning of the imaging patterns that are observed.

## 1. Introduction

Differentiating benign (inflammation, pancreatitis, ischemia, iatrogenic) and malignant biliary strictures is difficult despite the multiple available methods due to random sampling of tissue [1, 2]. Biopsy, cytological brushing, and needle aspiration have very low levels of diagnostic accuracy in this setting [3]. Thus, diagnosis and treatment of bile duct cancer is frequently delayed, requires operative intervention or long-term follow-up [4]. Probe-based Confocal laser endomicroscopy (pCLE) provides real-time histology and may be beneficial in diagnosing cases of indeterminate biliary strictures [1]. The principle of the imaging modality is to have focused light passed through a confocal aperture, thereby reducing scattered light above and below the plane [5]. Since only one single spot, the “confocal,” can be imaged at once, all light spots have to be scanned in the horizontal (and vertical) plane in order to have dynamic images [5]. To obtain a high contrast image, pCLE requires contrast injection such as fluorescein, which diffuses through the capillaries and stains the extracellular matrix of the surface epithelium [6]. The difference in contrast allows architectural analysis of the surface mucosa from neoplastic tissue [7]. By allowing confocal microscopic images of the GI mucosa to be collected during the endoscopic procedure, this modality has the potential to enable histologic diagnoses to be made in real time [1].

Pancreaticobiliary strictures are difficult to diagnose accurately because of limitations of current diagnostic methods and characteristics of malignancies [2, 8, 9] such as most of them grow along the bile duct wall [3, 6, 10–14]. The flexible probe-based confocal laser endomicroscopy (pCLE) has overcome many of the challenges met when evaluating the biliary tree [5]. Its small size allows for improved navigation through the small biliary tree and is flexible enough to be introduced even via small instrumentation channels, but rigid enough to enable exact placement and maneuverability of the probe tip [5]. For biliary imaging, the confocal miniprobe is passed through the channel of a side-viewing endoscope and advanced into the biliary tree through a hinged catheter or cholangioscope [4]. 

Applications of pCLE have proven feasible and clinically relevant in the case of examination of postendoscopic mucosal resection scars, polyps, and ulcerative colitis surveillance [6]. Recently, new pCLE criteria for the prediction of high-grade dysplasia/cancer in Barrett's esophagus patients were developed and validated [15].

Our study reviewed the limited studies evaluating indeterminate biliary strictures using pCLE and the histological basis for the confocal images [4].

## 2. Methods

**Figure 1:**
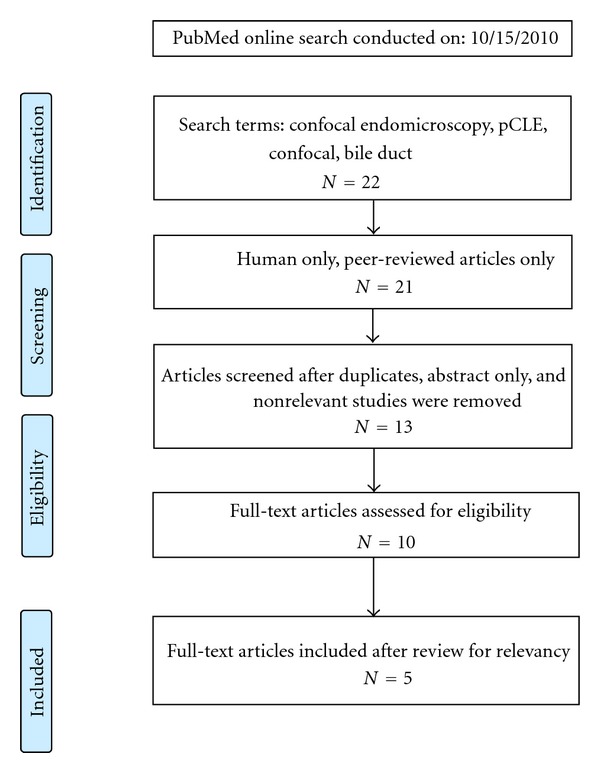
Literature review search schematic.

A PubMed search for relevant articles was performed using the following keywords: “pCLE”, “confocal”, “endomicroscopy”, “probe-based confocal laser endomicroscopy”, and “bile duct”. Peer-reviewed studies conducted between January 2000 and November 2011 were included. The search yielded a total of 22 articles, and after careful individual review for eligibility and relevancy, 5 were included in the final review (Figure 1).

### 2.1. Confocal Technique

CholangioFlex miniprobe has an outer diameter of less than 1.0 mm, a lateral resolution of 3.5 *μ*m, a focal plane 55 *μ*m beyond the probe tip, and 400 fold magnification [11]. It is compatible with a 1.2 mm operating channel, depth focus of 40 to 70 *μ*m, and a field view of 325 *μ*m [4].

GastroFlex^UHD^ miniprobe (UHDp) offers higher magnification and improved lateral resolution of 1 *μ*m but these probes are larger in diameter to accommodate an increased number of fiber optics [1]. It is compatible with a 2.8 mm working channel, depth focus of 55 to 65 *μ*m, and a field view of 240 *μ*m [4]. This probe however is not FDA approved for pancreaticobiliary applications; therefore, a confocal miniprobe is typically used to examine the biliary mucosa [4].

### 2.2. pCLE Technique

The CholangioFlex miniprobe is inserted into the CBD through a SpyGlass catheter or Olympus swing tip catheter [4]. The GastroFlex UHD miniprobe is inserted into the common bile duct (CBD) using a freehand technique typically alongside a 0.035-inch guide wire left in place [4]. Position of the probe was identified fluoroscopically. An injection of up to 10 mL of 0.5% to 10% fluorescein is then performed [4].

Depending on the indication, imaged areas were subsequently sampled by either brushing and/or biopsy forceps and sent for light microscopy [1]. Biopsy specimens were collected in 10% buffered formalin, routinely processed, paraffin embedded, and sectioned to be stained with H&E and examined using bright light microscopy [4].

## 3. Results

Meining et al'.s studies proposed imaging criteria for pCLE diagnosis of malignancy in indeterminate biliary strictures [5, 17]. Criteria include loss of reticular pattern of epithelial bands of less than 20 *μ*m; detection of irregular epithelial lining, villi, or glandlike structures; tortuous, dilated, and saccular vessels with inconsistent branching; and presence of “black areas” of more than 60 to 80 *μ*m (focally decreased uptake of fluorescein). Applying these hallmarks to 14 patients with biliary strictures (8 were benign and 6 were malignant), the sensitivity, specificity, and overall diagnostic accuracy for detection of neoplasia were 83%, 88%, and 86%, respectively. Comparatively, the sensitivity with traditional sampling methods was 50%. The study was performed utilizing the CholangioFlex miniprobe [1].

In [19] in 2012, a uniform classification of biliary and pancreatic pCLE findings (“miami Classification”) for indeterminate strictures was developed. The set of image interpretation criteria were tested through blinded consensus review of 112 randomized pCLE videos from 47 patients, and interobserver variability was assessed in 42 patients. The characteristics most suggestive of malignancy included the following: thick white bands (>* *20 *μ*m), or thick dark bands (>* *40 *μ*m), or dark clumps or epithelial structures. These provided sensitivity, specificity, positive predictive value, and negative predictive value of 97* *%, 33* *%, 80* *%, and 80* *% compared with 48* *%, 100* *%, 100* *%, and 41* *% for standard tissue sampling methods. Inter-observer variability was moderate for most criteria. Combining individual characteristics improved the sensitivity for the detection of malignancy [18].

In the work of Shieh et al., the GastroFlex^UHD^ miniprobe (UHDp) was successfully introduced into the CBD in 10 of 11 patients [1]. Cellular structures and individual cell morphology were more clearly visualized with the UHDp, as compared to the CholangioFlex probe. No significant side effects were exhibited except 1 case of mild pancreatitis [1].

Loeser et al. utilized the earlier published criteria by Meining et al. to evaluate possible malignancy in the pCLE images obtained during evaluation of 14 patients with indeterminate strictures [4]. pCLE determination was 6 with malignant strictures, 3 with benign strictures, and 5 with indeterminate strictures. The CholangioFlex miniprobe was used to image most biliary strictures and bile ducts, but occasionally the GastroFlex ultrahigh definition (UHD) was used [4]. This study found that the criteria were often nonspecific and found in both malignant and normal CBD. However, a normal reticular pattern without other markers of malignancy was seen in all normal patients [4]. Correlating the multiphoton microscopic study with the confocal images of rat's bile duct, it was found that the reticular network pattern seen is lymphatic structures [6]. There is some evidence that lymphangiogenesis occurs in cholangiocarcinoma, which might explain why the reticular pattern is distorted in this cancer [4, 10, 11, 19].

In the work of Giovannini et al., the CholangioFlex probe was used on thirty-seven patients (23 males) undergoing ERCP for bile duct stone removal (7 cases) or bile duct stenosis (30 cases) [16]. No complications occurred after the procedure and sufficient images were obtained in 33 patients. Final diagnoses were broken down as follows: normal CBD in 7 cases, 23 malignant stenoses (4 ampullary carcinomas, 13 cholangiocarcinomas, and 6 pancreatic cancer), and 7 inflammatory stenoses (4 chronic pancreatitis,1 stenosis of hepaticojejunal anastomosis, 1 postcholecystectomy CBD stenosis, and 1 primary sclerosing cholangitis) [16]. The pCLE images of a normal CBD showed a thin black band (<20 *μ*m) and normal vessels (thin and regular). Glands were not visible in benign cases. Malignant strictures displayed irregular vessels without contrast in the CBD wall, a large black band (>20 *μ*m), and irregular black cells (black clumps). These characteristics were seen in all malignant stenoses but were absent in benign or normal CBD. Neoplasia was predicted with an accuracy rate of 86%, sensitivity of 83%, and specificity of 75%. Standard histopathology had lower accuracy, sensitivity, and specificity, compared to pCLE. The values for biopsies were 53, 65, and 53% [16].

In a 2011 multicenter study, 89 patients, 61 of which had indeterminate biliary strictures, were evaluated using the CholangioFlex probe [9]. After one month of follow-up for patients with suspected malignancy, 40 patients were ultimately determined to have cancer [9]. pCLE had a sensitivity of 98%, specificity of 67%, and an accuracy of 81%, as compared to the index pathology values of 45%, 100%, and 75%. In addition to assessing the accuracy of pCLE, the accuracy of a cholangiopancreatoscopy versus a catheter for the pCLE insertion was evaluated; however a statistically significant result was not achieved. Interestingly, examiners reported increased confidence in procedure performance with time but did not show a statistically significant increase in specificity or sensitivity [9].

## 4. Discussion

**Figure 2:**
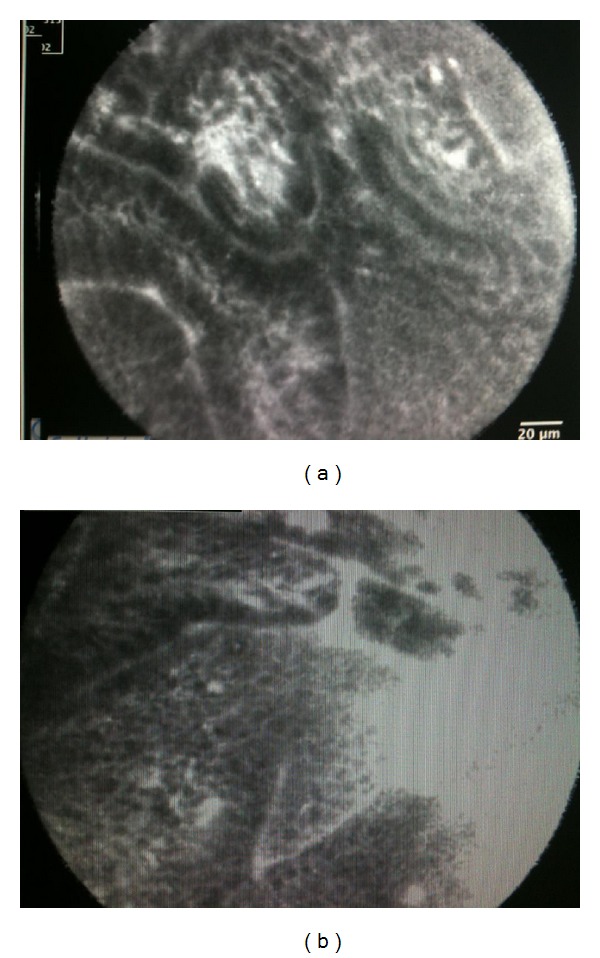
(a) Abnormal epithelium with decreased uptake of fluorescein in a patient with primary sclerosing cholangitis seen during a confocal procedure using a GastroFlex. (b) Abnormal epithelium with decreased uptake of fluorescein with loss of architecture integrity in a patient with primary sclerosing cholangitis seen during a confocal procedure using a GastroFlex.

**Figure 3:**
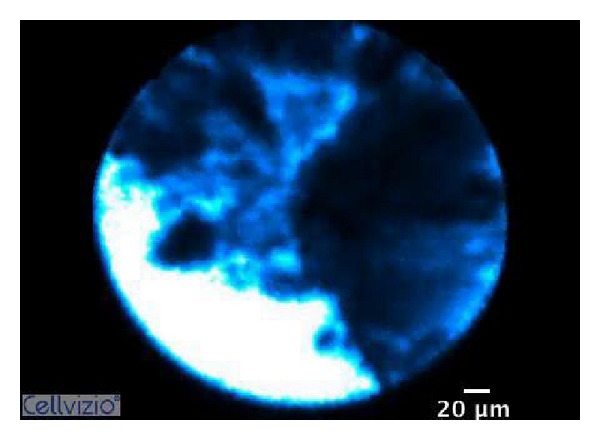
Dark clumps suggesting malignancy (according to Miami Classification) seen during a confocal procedure using a CholangioFlex.

**Figure 4:**
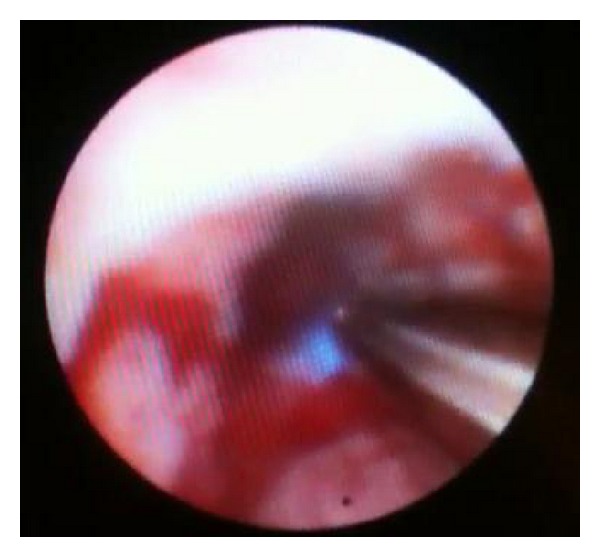
Cholangioscopy showing pCLE CholangioFlex probe being inserted into a bile duct.

Cholangiocarcinoma is particularly difficult to diagnose since its growth occurs along the bile duct wall rather than radially to form a mass [13, 14]. The small diameter of the pCLE fiber allows for easier and more accurate imaging of the mucosa and vessels within the biliary duct (Figure 2) [3, 4]. The major role of pCLE in the biliary tree is likely to detect malignancy in indeterminate bile duct and pancreatic strictures (Figures 3 and 4) [3].

Although Meining et al. and Loeser et al. suggest that certain imaging patterns provide an improved ability to distinguish among benign and malignant strictures, the histological significance of these patterns remains unclear [4]. Loeser et al. found some discrepancies with the published criteria developed by Meining et al. for the prediction of malignant biliary stricture [4, 6]. Noteworthy, in the work of Loeser et al., the negative predictive value could not be calculated in the study because of the small sample size of 14 [4]. Loeser et al. found that dilated blood vessels are present in the normal CBD and in cases of malignancy; therefore these criteria were not specific for malignancy [4, 6]. Due to these inconsistencies in early published criteria, Loeser et al. claimed that a negative pCLE study of the biliary tree is sufficient for ruling out a carcinoma. However, use of the earlier criteria can lead to numerous false positives [4]. Thus, the diagnostic criteria for pCLE in the biliary tree are still evolving and operating characteristics still need to be defined after the diagnostic criteria are settled upon [1].

It remains to be seen if accuracy could be improved through the use of the higher definition GastroFlex^UHD^ probe [1]. As the tip of the miniprobe should be perpendicular to the tissue surface to obtain the best image and so a smaller probe may be easier to manipulate with the limited space of the biliary system [1], thus, the easier manipulation of the smaller CholangioFlex miniprobe may come at the expense of image quality and spatial resolution [1].

Current data on pCLE in the bilary tree is promising yet sparse (Table 1). Also, malignant biliary strictures in the studies were not limited to cholangiocarcinoma but also included pancreatic metastatic cancers [4]. The irregular or abnormal vasculature noted in some of the studies could merely represent a paraneoplastic phenomenon [4]. Further studies are needed to ascertain how accurate pCLE can be for further differentiation of inflammation from malignancy [5]. A multicenter-registry has been initiated to further test the accuracy of the proposed hallmarks of pCLE and perhaps identify new criteria for malignancy versus inflammation [5]. Additionally, it remains unclear whether it is necessary to perform pCLE via cholangioscopes or whether positioning of the probes via standard ERCP catheters under fluoroscopic control is equally effective [5]. Lastly, the cost-effectiveness of pCLE should be assessed in comparison with other methods such as the fluorescence in situ hybridization technique [5].

## 5. Conclusion

Confocal endomicroscopy may provide a unique benefit by allowing in vivo, real-time histopathologic examination of several areas of interest within the biliary [1]. It is a novel and potentially promising modality for the biliary tree, although further studies must be conducted to establish its usefulness for the diagnosis of indeterminate biliary strictures and to understand the histological meaning of the imaging patterns that are observed [4].

Although pCLE is a valuable tool in the setting of indeterminate biliary strictures, it is important to note that the patient's clinical symptoms and abnormal cross-sectional imaging carry a pretest accuracy of the lesion/stricture being malignant in more than 85%. Thus although clinical symptoms and abnormal imaging prompt suspicion, diagnosis can be achieved through pCLE providing an imaging modality that can be coupled with real-time histological targeted biopsy [20]. More research data is needed to verify accuracy and determine pre- and posttest probability for malignant or benign biliary strictures.

The immediate impact of pCLE's modality in the biliary tree is the ability to target biopsies more precisely, while long-term impacts could include a rethinking of the dependence on histology as the biomarker of choice for detection, prognostication, and prediction of biliary disease and therapy [3].

## Figures and Tables

**Table 1:** Summary of study results.

Study	Date	Sample	No. malignant	Specificity pCLE	Sensitivity pCLE	Accuracy pCLE
Meining et al. [5]	2009	14	6	88	83	86
Shieh et al. [1]	2011	11	*	*	*	*
Loeser et al. [4]	2011	14	6	*	*	*
Giovannini et al. [16]	2011	37	23	75	83	86
Othman and Wallace [6]	2011	89	40	67	98	81

*Value not determined in study.
